# Supplementary material for: Designing ethical souvenirs to sustain the cultural integrity of Dunhuang heritage
Source: PLoS One. 2025 Dec 9;20(12):e0338506. doi: 10.1371/journal.pone.0338506 (PMC12688146; doi:10.1371/journal.pone.0338506)
Supplement: S1 Dataset — Thematic Analysis Codebook and RQ–Theme Mapping. This document contains all 59 codes derived from the thematic analysis, grouped into 8 categories and 3 themes with their descriptions, collectively addressing the two research questions. (DOCX) [file pone.0338506.s001.docx]

# S1 Dataset. Thematic Analysis Codebook and RQ–Theme Mapping

## Research Questions

RQ1. What are the fundamental ethical principles and values that guide designers in creating authentic souvenirs?

RQ2. How do designer philosophy and ethics impact cultural integrity in souvenir design?

Table 1 RQ-Theme Mapping

| **RQ** | **Theme** | **Category**  **(Sub-theme)** | **Code** | **Description** |
| --- | --- | --- | --- | --- |
| RQ2 | Design Principles and Philosophy | Culture-Oriented Design | Historical Authenticity | “It is crucial to showcase Dunhuang’s artistic splendor through tangible souvenirs, enabling the public to experience the magnificence of Mogao art.” |
|  |  |  | Cultural Symbolism |  |
|  |  |  | Material Selection |  |
|  |  |  | Traditional Techniques |  |
|  |  |  | Storytelling |  |
|  |  |  | Cultural Sustainability |  |
|  |  |  | Iconic Motifs |  |
|  |  |  | Regional Identity |  |
|  |  | Value of Aesthetics and Use | Visual Appeal | “The initial approach involves integrating traditional murals into items, such as blending the Protective Deities from Tianlong Babu with the popular and appealing blind box designs.” |
|  |  |  | Functionality Integration |  |
|  |  |  | User Experience |  |
|  |  |  | Market Appeal |  |
|  |  |  | Design Evolution |  |
|  |  |  | Consumer Interaction |  |
|  |  |  | Emotional Impact |  |
|  |  | Market-Centric Approach | Consumer Preferences | “It is crucial to plan product categories based on what customers like and what is useful.” |
|  |  |  | Product Utility |  |
|  |  |  | User Demand Analysis |  |
|  |  |  | Sales Trends |  |
|  |  |  | Consumer Feedback |  |
|  |  |  | Target Demographics |  |
|  |  |  | Market Segmentation |  |
|  |  |  | Purchasing Behavior |  |
|  |  |  | Product Affordability |  |
| RQ1 | Design Ethical Considerations | Ethical Boundaries and Cultural Sensitivity | Content Sensitivity | “From the perspective of design and production, it is necessary to avoid some not-so-good elements or contents.” |
|  |  |  | Moral Responsibility |  |
|  |  |  | Cultural Adaptation Strategies |  |
|  |  |  | Cultural Representation |  |
|  |  |  | Censorship Issues |  |
|  |  |  | Cross-Cultural Ethics |  |
|  |  |  | Designer Responsibility |  |
|  |  |  | Historical Sensitivity |  |
|  |  |  | Religious Considerations |  |
|  |  | Intellectual Property and Authorization | Public Domain IP | “The Mogao Cave murals are considered public intellectual property (IP), which means that their copyright has returned to the public domain.” |
|  |  |  | Copyright Issues |  |
|  |  |  | Licensing Agreements |  |
|  |  |  | Cultural Ownership |  |
|  |  |  | Reproduction Ethics |  |
|  |  |  | Trademark Considerations |  |
|  |  |  | Intellectual Property Rights |  |
|  |  |  | Cultural Symbolism in IP |  |
|  |  |  | Legal Challenges |  |
|  |  |  | International IP Laws |  |
|  |  | Regulatory and Ethical Constraints in Creative Adaptations | Subjective Influence Reduction | “However, as designers gradually accumulate experience and have a deeper understanding of the design content, the influence of personal subjectivity will decrease.” |
|  |  |  | Professional Ethics |  |
|  |  |  | Regulatory Compliance |  |
|  |  |  | Adaptive Design |  |
|  |  |  | Institutional Guidelines |  |
|  |  |  | Creative Boundaries |  |
|  |  |  | Designer Agency |  |
| RQ2 | Design Implementation | Iterative Adaptation | Market-Driven Adaptation | “We usually need several rounds of prototypes and modifications before we can settle on a final form.” |
|  |  |  | Prototype-first |  |
|  |  |  | In-store trial sale |  |
|  |  |  | Narrative-before-artifact |  |
|  |  |  | Crowdfunding/limited probe |  |
|  |  |  | Cross-functional routing |  |
|  |  | Practical Constraints in Market Alignment | Target price & MOQ envelope | “We usually need several rounds of prototypes and modifications before we can settle on a final form.” |
|  |  |  | Preserve core, simplify ancillary |  |
|  |  |  | Quality-in-hand trade-off |  |
