# Supplementary material for: Designing ethical souvenirs to sustain the cultural integrity of Dunhuang heritage
Source: PLoS One. 2025 Dec 9;20(12):e0338506. doi: 10.1371/journal.pone.0338506 (PMC12688146; doi:10.1371/journal.pone.0338506)
Supplement: Attachment — Submitted filename: Designing ethical.docx [file pone.0338506.s002.docx]

Review Comments

*Designing ethical souvenirs to sustain the cultural integrity of Dunhuang heritage*

A. General feedback:

This article necessitates significant amendments prior to publication; many of the citations are outdated, and considerable changes are required in the article's structure.

Based on these extensive and substantive comments, the article appears to require significant revisions to meet the standards for publication. The numerous issues—including outdated citations, structural organization, methodological clarity, and depth of analysis—suggest that it is currently not suitable for acceptance in its present form.

Therefore, the appropriate decision would be **reject**, with the recommendation that the authors thoroughly revise and resubmit after addressing all the outlined concerns.

B. Introduction:
1. The introduction must underscore the importance of ethical design practices that are directed towards the preservation of cultural integrity. Additionally, it should offer a more thorough elucidation of the existing research gap.
2. Many citations are missing or outdated, including but not limited to: “However, in recent years, the souvenir industry …..”, “Past research has explored various …..”, “The role of designers …...”, “Preserving cultural integrity in souvenir design …… “, and “Designers must prioritize cultural preservation over purely economic ….”
C. Literature Review and Theoretical Background:
1. Many citations are missing or outdated.
2. All citations are outdated.
3. In the Literature Review, establish the connection between Cultural Integrity and Design Principles and Philosophy, along with Design Ethical Considerations.
D. Methodology:
1. Add a sample size section and include:
   a. Justification for using “purposive sampling.”
   b. A discussion on sample size, further elaborating on “Theoretical saturation,” along with the recommended sample size for qualitative studies.
   c. The sample size for the pilot test is three; provide an explanation for this choice.
2. You indicated that the researcher received authorization from the University Ethics Committee, reference number to be added.
3. You indicated that “All participants volunteered to participate in the study, signed an informed consent form.” Please elucidate the contents of this form.
4. You mentioned, “The researcher was free of personal biases that could influence the research topic.” Was there any pre-test conducted to confirm this?
E. Results:
1. Please relocate the section pertaining to the development of themes to precede section 4, along with the corresponding subsections for each variable. Furthermore, the results, as represented in Figure 2 and Table 12, should be placed subsequent to the themes development section.
2. You have articulated how the interviews contributed to the development of codes and categories for two themes, namely, "Design Principles and Philosophy" and “Design Ethical Consideration". However, the interviews did not yield codes and categories for the theme “Design Implementation."
3. In the interviews, you also did not provide evidence or discuss how “Design Principles and Philosophy" and “Design Ethical Consideration” are connected to “Design Implementation."
4. In Sections 4.1 and 4.2, preceding the sub-sections, it is recommended to provide a comprehensive discussion on the primary theme, incorporating pertinent citations, and to appropriately introduce the sub-themes.
F. Discussion:

The discussion section shall emphasize the explanation of the Framework of Ethical Souvenir Design for Cultural Integrity and will link the results with recent studies.

G. Conclusion:

Include contributions: Theoretical, Practical (Emphasize recommendations based on categories and codes), and methodological.
