# Supplementary material for: Designing ethical souvenirs to sustain the cultural integrity of Dunhuang heritage
Source: PLoS One. 2025 Dec 9;20(12):e0338506. doi: 10.1371/journal.pone.0338506 (PMC12688146; doi:10.1371/journal.pone.0338506)
Supplement: Attachment — Submitted filename: Response_to_Reviewers plos one 1 round.docx [file pone.0338506.s004.docx]

# Response to Reviewers

**Manuscript Title: Designing Ethical Souvenirs to Sustain the Cultural Integrity of Dunhuang Heritage**

We sincerely thank the editor and reviewers for their thorough and constructive comments. We have carefully revised the manuscript in response to all points raised. Below we provide a detailed, point-by-point response.

A. General Feedback

| **Reviewer Comment** | **Response** | **Action in Manuscript** |
| --- | --- | --- |
| This article necessitates significant amendments prior to publication; many of the citations are outdated, and considerable changes are required in the article structure. The numerous issues—including outdated citations, structural organization, methodological clarity, and depth of analysis. | We appreciate the editor’s and reviewers’ insightful comments and have undertaken a comprehensive revision accordingly:  (1) Literature update: We replaced outdated citations with recent studies (2020–2025) and added cutting-edge work on emergent authenticity, commercialization tensions, place-based craft, and design ethics.  (2) Structural reorganization: We restructured the manuscript to improve logic and readability and sharpened the critical stance of the review.  (3) Methodological elaboration: We detailed purposive sampling and sample size rationale, reported code/meaning saturation, and clarified ethics approval and informed consent; we also tightened the evidence chain linking themes to data and literature.  (4) Data and results expansion: We added two interviewees to enhance trustworthiness, re-analyzed the dataset, and introduced one new theme aligned with the research framework.  (5) Discussion enhancement: We reorganized the discussion to mirror the (now three) themes, deepened theoretical interpretation, and articulated the EDCI framework with recent literature.  (6) Strengthened conclusion: We highlighted theoretical, practical, and methodological contributions and offered actionable design and governance recommendations. | √ Revisions applied **throughout the manuscript**: updated references across Introduction, Literature Review, and Discussion;  √ Reorganized **overall structure** for logical flow and readability;  √ Expanded **Methodology** (sampling, ethics, saturation);  √ Revised **Results** with additional data and one new theme;  √ Reorganized **Discussion** to mirror results and deepen theoretical interpretation;  √ Strengthened **Conclusion** with explicit theoretical, practical, and methodological contributions. |

B. Introduction

| **Reviewer Comment** | **Response** | **Location in Manuscript** |
| --- | --- | --- |
| The introduction must underscore the importance of ethical design practices for preserving cultural integrity and more clearly articulate the research gap. | Reframed the opening to foreground cultural integrity as a non-negotiable design constraint (UNESCO/ICOMOS anchor). | 1. Introduction, pp. 2–3. Line 34-48. |
|  | Rewrite a focused research gap: … Most prior studies treat cultural preservation and design ethics as separate lines of inquiry, without exploring the mechanisms through which designers’ moral principles and decision-making frameworks and cultural responsibilities influence the creation of authentic heritage products. | 1. Introduction, pp. 3–4. Line 49-61. |
| Many citations are missing or outdated, including but not limited to: “However, in recent years, the souvenir industry …..”, “Past research has explored various …..”, “The role of designers …...”, “Preserving cultural integrity in souvenir design …… “, and “Designers must prioritize cultural preservation over purely economic ….” | In the revised Introduction, we rewrote the flagged generic statements and replaced outdated sources with recent, authoritative references. Specifically, we  (1) anchored “preserving cultural integrity” in international norms and provided a study-specific working definition (UNESCO/ICOMOS; see [1]–[4]);  (2) substantiated the “in recent years/past research” claims with up-to-date syntheses and empirical studies on souvenirs, authenticity, commercialization, and sustainability (see [5]–[10], [16]–[20], [22]);  (3) reframed the “role of designers” as an ethics-based responsibility supported by design-ethics literature (see [11], [12]) rather than a declarative assertion; and  (4) removed or merged legacy/duplicative citations to keep the narrative current and coherent. These revisions ensure that all key statements in the Introduction are evidence-based, contemporary, and aligned with the paper’s theoretical framing. | 1. Introduction, pp. 3–4. Line 35-68. |

C. Literature Review and Theoretical Background

| **Reviewer Comment** | **Response** | **location in Manuscript** |
| --- | --- | --- |
| 1. Many citations are missing or outdated. | In the revised Literature Review, we systematically updated the references, replacing outdated citations with recent and authoritative sources (2018–2025). This ensures that the discussion reflects the current state of research on souvenirs, cultural sustainability, and design ethics. | 2. Literature Review, pp.4-8，line 82-167. |
| 2. All citations are outdated. | We carefully re-examined all references and replaced older sources with up-to-date publications. In addition, we retained only a small number of classic works that provide historical grounding, while ensuring that the majority of citations now come from recent studies. | 2. Literature Review, pp.4-8，line 82-167. |
| 3. Establish the connection between Cultural Integrity and Design Principles/Philosophy, along with Design Ethical Considerations. | We restructured the section titles to make the conceptual progression explicit (2.1 Cultural Integrity in Heritage Product Design; 2.2 Design Principles and Philosophy for Ethical Souvenirs; 2.3 Design Ethical Considerations in Commercializing Heritage; 2.4 Conceptual Framework for Ethical Heritage Souvenir Design). |  |
|  | We also rewrote the content to highlight the linkages: 2.1 concludes by emphasizing the need to bridge heritage values with contemporary design concepts; 2.2 defines design philosophy and principles as actionable guidelines reinforcing cultural integrity; 2.2 transitions into 2.3 by pointing out that applying these principles raises ethical issues, which are elaborated through virtue ethics, value-sensitive design, and participatory approaches; finally, 2.4 synthesizes the three strands into a conceptual framework that positions cultural integrity, design philosophy/principles, and ethical considerations as interdependent components ensuring both authenticity and market viability. These revisions provide a coherent theoretical foundation and directly address the reviewer’s request. |  |

D. Methodology

| **Reviewer Comment** | | **Response** | **Location in Manuscript** |
| --- | --- | --- | --- |
| 1. Add a sample size section and include: | a. Justification for using “purposive sampling.” | We added a detailed explanation of purposive sampling. A two-tier purposive sampling strategy was described: (i) case selection of the Dunhuang Academy product development sector based on four representativeness criteria; (ii) participant selection with three criteria (professional role, ≥1 year relevant experience, direct engagement with Dunhuang cultural elements). This clarified why purposive sampling was necessary to capture information-rich participants with heritage-specific expertise. | 3. Methodology, pp. 9-10, line 173-188. |
|  | b. A discussion on sample size, further elaborating on “Theoretical saturation,” along with the recommended sample size for qualitative studies. | In the revised manuscript, we increased the number of interviews from 12 to 14, comprising 3 pilot interviews and 11 main interviews. The sample now includes 8 designers and 3 cultural experts (previously 7 designers and 2 cultural experts). Following reviewer suggestions, we added two participants—one designer and one expert—to enhance thematic saturation and the rigor of the theoretical framework.  “Data analysis was subsequently performed, supplemented by dual criteria for descriptive saturation: (i) code saturation, whereby no new codes emerged across two consecutive interviews; and (ii) meaning saturation, when sufficient richness and variation had been captured to delineate the properties of existing codes [63]. These criteria were satisfied after the ninth interview. To ensure stability and confirm the robustness of the findings, two additional interviews were conducted, which produced no new codes or themes [64].” | 3. Methodology, pp. 10, line 193-201.  3. Methodology, pp. 12, line 219-227. |
|  | c. The sample size for the pilot test is three; provide an explanation for this choice. | In the revised manuscript, we explained that the rationale for conducting the pilot study with three participants aligns with standard practice for optimizing interview protocols through small-scale qualitative research.  We state: “A total of 14 interviews were conducted, including three additional interviews with designers as a pilot study to test content validity, clarity of wording, sequencing, timing, and logistics of the interview protocol. In qualitative inquiry, small pilots (≈2–5) are standard because the purpose is protocol refinement rather than saturation or theory building [56,57]. Insights from the pilot led to minor revisions to the interview questions; pilot data were not included in the final analysis.” | 3. Methodology, pp. 10, line 199-204. |
| 2. You indicated that the researcher received authorization from the University Ethics Committee, reference number to be added. | | We included the official ethics approval reference number (LUFE-2022-0721) in the methodology section and clarified that follow-up interviews were conducted under the same approval with an approved extension. | 3. Methodology, pp. 12, line 231. |
| 3. You indicated that “All participants volunteered to participate in the study, signed an informed consent form.” Please elucidate the contents of this form. | | We detailed the informed consent form contents:  “All participants volunteered to participate in the study and signed an informed consent form, were treated with total respect during the investigation, and were allowed to check all documents concerning them. The form included (i) the purpose and procedures of the study; (ii) the voluntary nature of participation; (iii) the right to withdraw at any stage without penalty; (iv) assurance of anonymity and confidentiality; and (v) agreement for audio recording and use of data solely for academic purposes.” | 3. Methodology, pp. 12, line 232-237. |
| 4. You mentioned, “The researcher was free of personal biases that could influence the research topic.” Was there any pre-test conducted to confirm this? | | We expanded this section to explain the reflexive measures taken:  “The researcher was free of personal biases that could influence the research topic [66]. As researchers independent of the profession, their potential biases were addressed through continuous reflexivity and qualitative-appropriate strategies. Before and throughout data collection, a reflexive journal was maintained to document assumptions, positionality, and evolving reflections [67]. In addition, a pilot interview was used to refine questions, and triangulation, member checking, trail audits, and peer feedback further helped mitigate predispositions. These practices allowed the researcher to critically examine personal assumptions and minimize their influence on data collection and interpretation.” | 3. Methodology, pp. 12-13, line 241-248. |

E. Results

| **Reviewer Comment** | **Response** | **Location in Manuscript** |
| --- | --- | --- |
| 1. Please relocate the section pertaining to the development of themes to precede section 4, along with the corresponding subsections for each variable. Furthermore, the results, as represented in Figure 2 and Table 12, should be placed subsequent to the themes development section. | We have added a new opening paragraph in Section 4 (Results) to present the theme-development process up front. The paragraph now reads:  “A total of 59 initial codes were generated from the interview transcripts and supplementary documents. Through iterative clustering and abstraction, these codes were consolidated into eight categories and three overarching themes: (1) Design Principles and Philosophy, (2) Design Ethical Considerations, and (3) Design Implementation. Following a reflexive thematic analysis, coding decisions were repeatedly revisited to ensure transparency and conceptual rigor. The progression from raw data to thematic abstraction is illustrated in Fig 3. Design principles and philosophy establish the cultural anchors and set out how Dunhuang motifs are translated into aesthetic and functional product terms. Design ethical considerations provide the boundary conditions and procedures—cultural sensitivity, authorization, and intellectual property—that moderate these choices and ensure legitimacy. Design implementation operationalizes the resulting decisions through prototyping, trial exposure, and revision under cost, material, and process constraints, and the evidence it generates feeds back to refine both principles and ethical rules. In combination, the three themes form an iterative system in which anchoring, governance, and execution co-produce cultural integrity and market relevance. Table 2 summarizes the final thematic framework with representative quotations.”  We also moved the results displays to follow this development. The former Figure 3 and Table 2 now appear after this paragraph. | 4. Results (theme development added), pp. 14–15, line 262-276. |
| 2. You have articulated how the interviews contributed to the development of  codes and categories for two themes, namely, Design Principles and Philosophy; and “Design Ethical Consideration. However, the interviews did not yield codes and categories for the theme “Design Implementation.” | We re-analyzed the interview corpus (including the two additional interviews added under the same ethics approval) and now present explicit codes and categories for “4.3 Design Implementation.” The new evidence shows how designers operationalize principles and ethics in practice. We introduced the two categories under this theme: 4.3.1 Iterative Adaptation and 4.3.2 Practical Constraints in Market Alignment. | 4. Results, pp. 14-15, line 706, Table 2.  4. Results, pp. 26–28, line 982-1031. |
| 3. In the interviews, you also did not provide evidence or discuss how “Design Principles and Philosophy; and “Design Ethical Consideration” are connected to “Design Implementation. | We added content on how “Principles/Philosophy” & “Ethical Considerations” connect to “Implementation.”  1. The cross-theme mechanism is stated upfront (4. Results opening paragraph):  “Design principles and philosophy establish the cultural anchors and set out how Dunhuang motifs are translated into aesthetic and functional product terms.”  “Design ethical considerations provide the boundary conditions and procedures—cultural sensitivity, authorization, and intellectual property—that moderate these choices and ensure legitimacy.”  “Design implementation operationalises the resulting decisions through prototyping, trial exposure, and revision … and the evidence it generates feeds back to refine both principles and ethical rules.”  “In combination, the three themes form an iterative system in which anchoring, governance, and execution co-produce cultural integrity and market relevance.” | 4. Results, pp. 14–15, line 262-276. |
|  | 2. Add two paragraphs under 4.3 Design implementation to link the other two themes:  The first paragraph: “This theme shows how the philosophical and ethical orientations identified earlier are translated into concrete practice. Implementation unfolds through continuous iteration and through decisions shaped by materials, processes, cost, and market fit.”  The last paragraph: “Design implementation is the practical process of validating the feasibility of design principles and philosophical frameworks within real-world constraints. It guides designers to respect cultural boundaries, transform Dunhuang heritage into tangible souvenirs, secure authorization of usage rights, and comply with regulatory norms to prevent misuse. In this way, the implementation phase bridges the concept-driven innovation with ethical responsibility, which reflects an iterative process of creative intent, review, and market adaptation. This interconnection demonstrates that cultural authenticity and consumer appeal can be maintained when philosophy, ethics, and practice operate in tandem.” | 4. Results, pp. 26, line 492-494.  4. Results, pp. 28, line 533-540. |
|  | 3. We elaborated on the link of the three themes in 4.4 A Framework of Ethical Souvenir Design for Cultural Integrity. | 4. Results, pp. 28-29, line 541-555. |
|  | 4. We also updated display Fig. 3 (codes → categories → themes) and Table 2 (thematic map with quotes) and Fig 6 (Framework of Ethical Design for Cultural Integrity) to show the linkage between three themes. | 4. Results, pp. 15-16, line 276-280, Fig. 3 and Table 2.  4. Results, 4.4 pp. 28, line 543 |
| 4. In Sections 4.1 and 4.2, preceding the sub-sections, it is recommended to provide a comprehensive discussion on the primary theme, incorporating pertinent citations, and to appropriately introduce the sub-themes. | Add supplementary content to Section 4.1 Design Principles and Philosophy to provide a comprehensive discussion, incorporate relevant citations, and appropriately introduce subtopics:  “Design principles guide both the symbolic and practical dimensions of product creation, shaping authenticity, usability, and market relevance [71]. In the innovation of Dunhuang souvenirs, these principles represent the normative foundations through which cultural products are conceptualized and positioned. They structure the translation of cultural narratives into material form and reveal three interrelated orientations: embedding cultural identity within design, integrating aesthetic qualities with practical use, and aligning production with market expectations. These orientations illustrate how designers negotiate the tension between heritage preservation and consumer appeal, thereby linking design philosophy to the maintenance of cultural integrity.” | 4. Results, pp. 16-17, line 284-292. |
|  | Add supplementary content to Section 4.1.1 Culture-oriented Design to begin a comprehensive discussion:  “Culture-oriented design advocates embedding local heritage and symbolic meaning in product form, ensuring that commercial souvenirs narrate local culture.” | 4. Results, pp. 17, line 294-295. |
|  | Add supplementary content to Section 4.1.2 Value of Aesthetics and Use to begin a comprehensive discussion:  “Aesthetic appeal and practical functionality shape consumer perception and long-term engagement, as designers aim to merge visual attraction with everyday usability.” | 4. Results, pp. 19, line 352-353. |
|  | Add supplementary content to Section 4.1.3 Market-Centric Approach to begin a comprehensive discussion:  “Market-centric design reflects a strategy of matching product categories with consumers' desire for cultural integrity.” | 4. Results, pp. 21, line 388-389. |
|  | Add supplementary content to Section 4.2 Design Ethical Considerations to provide a comprehensive discussion, incorporate relevant citations, and appropriately introduce subtopics:  “Design principles establish guidelines for shaping the core values of cultural products, which link to ethical responsibilities in practice. Ethical considerations serve as a moral compass, guiding the process of transforming Dunhuang heritage into authentic souvenirs. These considerations include respect for cultural boundaries, appropriate authorization of usage rights, and compliance with broader regulatory norms. The sub-themes represent the tension between creative freedom and cultural responsibility, reminding designers to innovate products that embody cultural dignity.” | 4. Results, pp. 22-23, line 408-414. |
|  | Add new theme 4.3 Design Implementation to support the theoretical framework. | 4. Results, pp. 26-28, line 491-540. |

F. Discussion

| **Reviewer Comment** | **Response** | **Location in Manuscript** |
| --- | --- | --- |
| The discussion section shall emphasize the explanation of the Framework of Ethical Souvenir Design for Cultural Integrity and will link the results with recent studies. | In the revised manuscript, we restructured and expanded the Discussion section to emphasize the Framework of Ethical Souvenir Design for Cultural Integrity. Specifically:  1. We rewrote the Discussion as three new subsections: 5.1 Design Principles and Philosophy for Preserving Cultural Integrity, 5.2 Design Ethics as Boundary Conditions for Innovation, and 5.3 Design Implementation within Ethical Constraints, which correspond directly to the framework’s three components (Design Principles and Philosophy, Design Ethics, and Design Implementation).  2. Each subsection now explains how its respective component functions within the framework, linking empirical findings to conceptual roles.  3. The revised text clarifies how the framework demonstrates the iterative interplay of cultural anchoring, ethical governance, and practical implementation in producing souvenirs that preserve cultural integrity while ensuring market relevance. | 5. Discussion, pp. 29–33, line 565-650. |
|  | Each subsection referred to the findings in recent scholarship: 5.1 links authenticity/affect/designerly reasoning to [72–76]; 5.2 connects procedural ethics, design justice, and cultural/IP rights to [35,42,77–79]; 5.3 relates iterative implementation and constraint-driven refinement to [80–82]. This provides an up-to-date, evidence-based discussion aligned with the results. |  |

G. Conclusion

| **Reviewer Comment** | **Response** | **Location in Manuscript** |
| --- | --- | --- |
| Include contributions: Theoretical, Practical (Emphasize recommendations based on categories and codes), and methodological. | The Conclusion has been rewritten to explicitly present all three contribution types:  6.1 Theoretical contribution.  6.2 Practical contribution (derived from categories/codes). We translate the thematic findings into actionable recommendations directly based on the categories and codes, including: Design-principle level, Ethics level, and Implementation level.  Methodological contribution (made explicit in the 6. Conclusion opening paragraphs). | 6. Conclusion, pp. 33–35, line 652-691. |

H. Other revision

In addition to the major changes requested, we also refined the language for clarity and conciseness, updated figures and tables to improve alignment with the revised results and discussion. We corrected minor inconsistencies in terminology, referencing, and formatting throughout the manuscript. These adjustments ensure that the paper meets both scholarly standards and readability expectations.

Thank you very much again.
